# What is the role of the primary care system in the initial management of psychotrauma?

**DOI:** 10.1192/j.eurpsy.2023.2075

**Published:** 2023-07-19

**Authors:** B. Zineb, T. Aicha, K. Imane, L. Fouad, O. Abderrazak

**Affiliations:** 1 Faculty of Medecine and Pharmacy of Rabat, Ar-razi Psychiatric Hospital, Rabat, Morocco

## Abstract

**Introduction:**

Post-traumatic stress disorder is common and the risk of developing it after a trauma is high. Its global management is long and complex.

The general practitioner, as a primary care provider, has a fundamental role in detecting it. They may have to take care of these patients both physically and psychologically, and may find themselves at a loss when faced with repercussions such as Post-Traumatic Stress Disorder (PTSD).

In some countries, the role of the general practitioner is different. More involved and better trained, they are actors in the pre-hospital system in case of psychotrauma.

**Objectives:**

By means of a survey conducted among general practitioners in Morocco, we will first try to determine their place in the screening and management of psychotrauma, and then to evaluate their knowledge of EMDR therapy.

**Methods:**

For this purpose, a questionnaire was developed and used as a basis for the study.

It included practical questions relating to the physicians’ activity, their practice in emergency consultations, their training, their management of physical and psychological trauma, and finally their knowledge of EMDR therapy,

Finally, we will attempt to propose concrete avenues for more effective, safer, and non-harmful management of psychotrauma in the basic health care system.

**Results:**

the general practitioner was the first professional to receive a patient who was a victim of a physical or psychological trauma (road accident, rape, physical or verbal aggression), but the degree of knowledge and training for this type of care was low among our doctors.

**Conclusions:**

Training sessions should be scheduled for general practitioners in order to optimize the management and prevention of post-traumatic stress disorder.

**Disclosure of Interest:**

None Declared

